# Treatment of Geriatric Acetabular Fractures Using the Modified Stoppa Approach: A Review Article of Technique, Tips, and Pitfalls

**DOI:** 10.3390/jcm13195867

**Published:** 2024-10-01

**Authors:** Mikolaj Bartosik, Eckart Mayr, Ulf Culemann

**Affiliations:** 1 Department of Trauma Surgery, General Hospital Celle, 29223 Celle, Germany; 2 Department of Orthopaedics, General Hospital Celle, 29223 Celle, Germany

**Keywords:** acetabulum, fractures, injuries, aged, geriatric, osteosynthesis, trauma surgery

## Abstract

The surgical treatment of geriatric acetabular fractures is becoming increasingly important due to a demographic change in age. While acetabular fractures used to occur more frequently in younger patients, they are currently more prevalent in geriatric patients. This change has also led to an increase in the frequency of anterior and combined anterior acetabular fractures. Surgery for geriatric acetabular fractures is very challenging, and surgeons need years of experience to be able to deal with the advantages and disadvantages of pelvic surgery. This is why a high level of surgical expertise is required. The aim of this article is to provide an insight into the topic of geriatric acetabular fractures with a critical narrative review of the current literature and a focus on minimally invasive surgical treatment using the modified Stoppa approach without patients’ own assessment. The modified Stoppa approach offers excellent visibility of the anterior structures of the acetabulum and can address the quadrilateral surface effectively. Pelvic surgery, in particular acetabular surgery, offers patients many advantages, such as rapid mobilization and the quick relief of pain symptoms. Total hip arthroplasty is currently being discussed as an alternative with good results for certain types of acetabular fractures in older patients, though it requires clarification of studies and recommendations.

## 1. Introduction

Acetabular fractures in geriatric patients are becoming increasingly important for orthopedic and trauma surgeons. The increasing age of the population, the decrease in bone density associated with age, and the desire to remain active and mobilized in elderly patients are leading to an increasingly frequent occurrence of geriatric acetabular fractures [1,2].

While a few decades ago conservative therapy was recommended in all elderly patients with an acetabular fracture [3], today surgical treatment is favored when indicated in order to preserve mobility and ensure a quick return to life participation [4,5]. In addition, it has been shown that surgical therapy is associated with better clinical outcomes, especially with displaced acetabular fractures [6,7,8]. If indicated, geriatric acetabular fractures should be treated surgically as soon as possible after the fracture event, since the outcome may deteriorate and reduced mobility leads to increased mortality and, thus, a poor prognosis [9].

In geriatric patients, acetabular fractures are often localized in the anterior structures [1,2,10]. Rapid mobilization is achieved using minimally invasive approaches for the anterior structures of the acetabulum, such as the modified Stoppa approach or the pararectus approach. These approaches have shown excellent clinical results in the treatment of elderly patients with acetabular fractures [11,12,13]. Nevertheless, the choice of approach is based on the localization of the fracture and the fracture’s morphology, thus its classification.

In this critical literature review, we would like to present important aspects of geriatric acetabular fractures and their surgical treatment using the modified Stoppa approach without patients’ own assessment.

## 2. Epidemiology

In the past, acetabular fractures occurred more frequently in young patients with the corresponding trauma, whereas, today, an increasing number of acetabular fractures are occurring in elderly patients, forming the majority [1,14].

In Europe, several independent studies have shown an increasing incidence of geriatric acetabular fractures. In a large register study from Germany, it was found that 50.5% of patients were over 60 years old, with an average age of 76.6 ± 9.5 years [1]. In Sweden, there was an increase in the incidence of acetabular fractures from 8.7 to 11 per 100,000 persons/years between 2001 and 2016, with a clearly greater growth in incidence in elderly patients [15]. Rinne et al. confirmed the trend and showed an increased incidence of geriatric acetabular fractures in patients >65 years of age from 17/100,000/persons/year to 23/100,000/year [16]. The French population also reflected the increase in patients >75 years of age from 17.06 to 23.18 per 100,000 persons/years [17].

Furthermore, this trend was confirmed for the United States by Ferguson et al., with a 2.4-fold increase in the incidence of geriatric acetabular fractures in the period from 1980 to 2007 [2]. This trend is confirmed in numerous studies [10,18,19]. As a result of this development, surgical treatment and recommendations have changed. While, in the 20th century, Letournel himself described not necessarily operating on patients over 60 years of age with an acetabular fracture [3], especially in cases of a poor bone quality, a distinctly surgical approach has emerged since the late 20th century and early 21st century [8,20].

A major problem with the increasing age of patients is the decreasing bone density, coupled with gait instability, which can lead to a fall and, thus, an acetabular fracture. As the number of geriatric patients increases, so does the frequency of fractures involving anterior acetabular structures [1,2,10,18].

**Figure 1:**
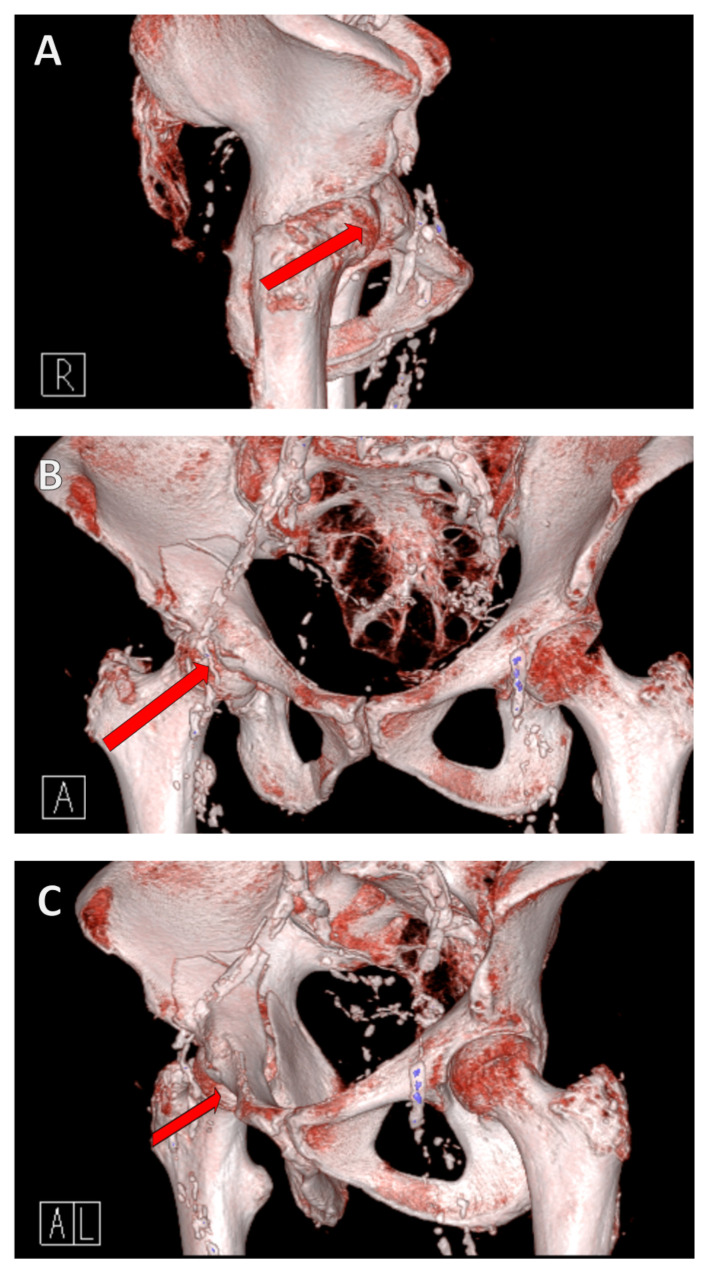
**Fracture mechanism of a geriatric acetabular fracture.** A fall from a standing position to the side results in a transfer of force from the greater trochanter via the femoral neck to the femoral head, where the force is then transferred to the anterior superior into the acetabulum. A reduced bone density causes the acetabulum to collapse/fracture. The red arrow in the illustration shows the force trajectory, from the right lateral (**A**), anterior (**B**), and left anterolateral views (**C**).

The mechanism of the fracture is as follows: a fall from a standing position causes a lateral fall onto the hip. This results in a transfer of force from the greater trochanter via the femoral neck to the femoral head, where the force is then transmitted to the anterior superior in the acetabulum itself due to the femoral anatomy (Figure 1).

## 3. Classification

**Figure 2:**
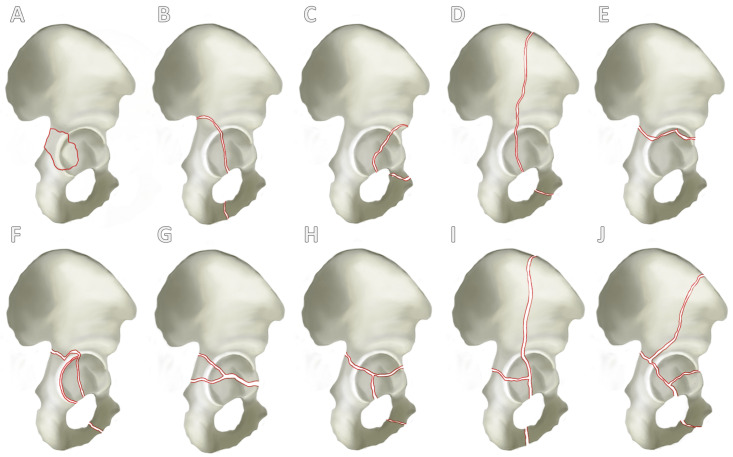
**Letournel’s classification.** A differentiation is established between 5 simple fracture types and 5 combined fracture types. Simple fracture types (**A–E**) are the fracture of the posterior acetabular rim (**A**), posterior pillar (**B**), anterior acetabular rim (**C**), anterior pillar (**D**) and the transverse fracture (**E**). A further distinction can be seen between combined fracture types (**F–J**): fracture of the posterior pillar and posterior acetabular rim (**F**), transverse fracture and involvement of the posterior acetabular rim (**G**), T-fracture (**H**), anterior pillar with hemivertebral fracture (**I**) and the two-pillar fracture (**J**).

There are currently several classifications for the categorization of acetabular fractures, whereby it can be said that the classification according to Judet and Letournel from 1980 forms the standard in orthopedic and trauma surgery [21,22]. Judet and Letournel classified acetabular fractures into 10 types of fractures according to localization and fracture extension. These were divided into five simple fractures (Figure 2A–E) and a further five combined fractures of different structures of the acetabulum (Figure 2F–J).

Another often used classification model was introduced by the AO Foundation (German acronym for “Arbeitsgemeintschaft Osteosynthese”)/Orthopedic Trauma Association (AO/OTA) in 1996 [23]. This classification model is based on that of Letournel and, therefore, considers the columns and walls of the acetabulum. However, one major difference is that articular involvement plays a major role in the subdivision: 62A, partial articular involvement with isolated fracture of a column and/or wall; 62B, partial articular involvement with transverse fracture; and 62C, complete articular involvement with fracture of both columns. A further distinction is then made depending on the course of the fracture, resulting in a total of 20 subtypes of acetabular fractures (Table 1).

An advantage of the AO/OTA classification is the more precise categorization, which enables a more accurate differentiation. Furthermore, the assessment of the joint surface also allows statements to be made about the possible outcomes. Fractures with complete joint involvement show a significantly worse outcome with an increased risk of joint arthrosis [24]. However, the categorization into up to 20 different subtypes is very prone to error and could lead to frequent mistakes by colleagues with less experience.

Periprosthetic acetabular fractures are classified separately, as cup stability is a key component of surgical planning [25]. The most common classification of periprosthetic acetabular fractures is the classification by Paprosky and Della Valle from 2003 [26].

## 4. Diagnostics

**Figure 3:**
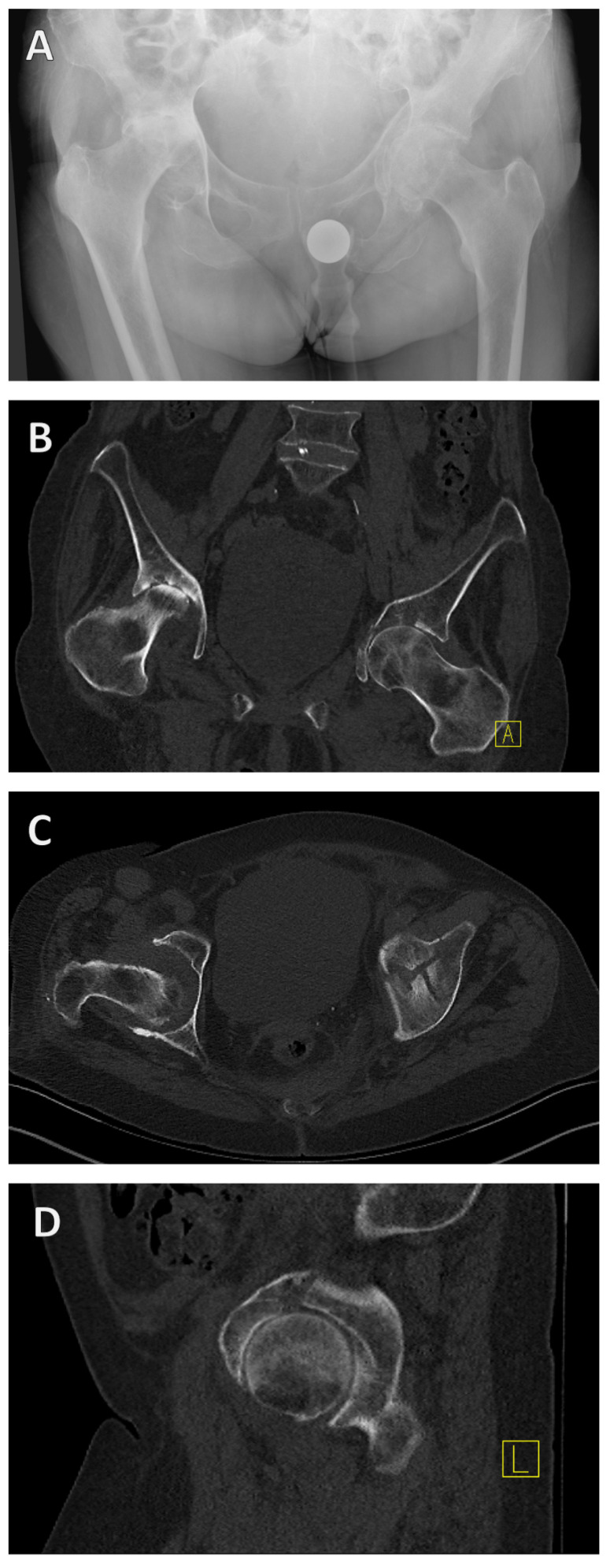
**X-ray and CT diagnosis of a geriatric acetabular fracture.** Initially, the left-sided acetabular fracture can be recognized in the anterior–posterior radiograph of the pelvis (**A**). A CT scan was added for fracture classification and preoperative planning (**B**–**D**). It revealed a T-shape fracture (**B**–**D**).

Alongside medical history and physical examination, radiological imaging techniques play a key role in the diagnosis of acetabular fractures. Both X-ray examination in the anterior–posterior, ala, and obturator beam of the pelvis and CT are used as standard procedures and are highly recommended [27] (Figure 3).

If an acetabular fracture cannot be ruled out with certainty in the X-ray examination, a CT scan should be performed. CT is not only used to confirm the diagnosis but also to categorize the fracture as described above (Figure 3B–D). CT can therefore be used to categorize the fracture and plan the surgical treatment. It is important to know the classification, as the approach is determined in the planning phase after the diagnosis and categorization.

Other radiological methods such as magnetic resonance imaging (MRI) are not suitable as they can primarily be used to assess the soft tissue situation, which plays a subordinate role in fracture stability at this point. Scintigraphy is also unsuitable in the postoperative phase.

**Treatment of geriatric acetabular fracture**

Both conservative and surgical treatment options are available for elderly patients. Both treatment options show a relatively high 1-year mortality rate of up to 25%, whereby the mortality rate does not differ significantly according to Wollmerstädt et al. [28]. Nevertheless, Firoozabadi et al. were able to show an improved 1-year mortality rate of 12% (versus 44%) in surgically treated geriatric patients with an acetabular fracture compared to those treated conservatively [5].

Surgical therapy enables patients to more rapidly bear weight, become mobile, and regain their independence [7]. Nevertheless, conservative treatment can be attempted for minimally displaced fractures, as there is no significant difference in outcome in these cases [29].

However, surgery-specific complications should be taken into account, and the risks of anesthesia should also be considered with an increasing age. Following surgery, up to 23.1% of patients may still require further surgery, specifically total hip arthroplasty [30].

## 5. Conservative Therapy

Conservative therapy used to be recommended for all patients > 60 years of age with an acetabular fracture [3]. Currently, decisions are made on an individual basis depending on the patient’s age, comorbidities, and fracture type. In geriatric patients, it is important to achieve mobilization as early as possible, as, otherwise, the risk of mortality may increase with immobility.

Conservative treatment is indicated in geriatric acetabular fractures if the fracture is not or only moderately displaced (≤2 mm) or for stable fractures without involvement of the hip joint and without involvement of the roof arc [6,31,32]. Furthermore, conservative treatment is indicated for an intact weight-bearing dome, a stable joint without the tendency to dislocate, fractures >3 weeks old, and geriatric patients with many comorbidities who cannot withstand the risk of surgery and the corresponding anesthesia or do not wish to undergo surgical treatment.

Quick mobilization with physiotherapy is recommended, initially in the bed and in a sitting position, followed by reduced weight bearing for the first 6–8 weeks [33]. It is important to avoid strict immobilization to reduce potential complications [8].

However, the early surgical treatment of acetabular fractures in older patients showed a significantly better clinical outcome [7,20]. Differences in mortality are not conclusively confirmed [34,35].

## 6. Surgical Therapy

Geriatric patients often require rapid mobilization, which is why surgical therapy can be advantageous, as it can achieve the quickest possible mobilization and a better clinical outcome [7]. On the other hand, it was found that patients can experience significant clinical deterioration following surgical treatment, despite good surgical care [36].

Surgical therapy is indicated if the fracture is displaced ≥2 mm, the hip joint is unstable, the hip joint is incongruent, intra-articular fragments are present, there is nerve damage, or a secondary fracture collapse occurs.

Since the most common geriatric acetabular fractures are fractures of the anterior acetabular structures, approaches that address these structures are of great relevance. One of these approaches is the modified Stoppa approach, which we will present in detail below.

The different anatomy of the pelvis or pelvic asymmetry [37] in different populations but also between men and women can be addressed by preoperative planning. With new implants, such as the DePuy Synthes 3.5 mm Intrapelvic Acetabular Plates, this individual anatomy can be addressed by choosing between three different sizes and bending the plate according to the acetabulum.

This approach has been shown to be superior to the ilioinguinal approach in the treatment of geriatric acetabular fractures in terms of operating time, blood loss, and complication rates in elderly patients [18,20]. However, there is no significant difference in the clinical outcome of the two approaches [20]. Also, the modified Stoppa approach is technically demanding, and, in its minimally invasive design without the first window of the ilioinguinal approach, it should only be chosen with appropriate surgical experience. Otherwise, the risk of complications such as retroperitoneal bleeding (mostly venous from the transition of the dorsal obturator vein into the external iliac vein) and nerve injuries in the area of the obturator nerve is drastically increased.

General complications of the modified Stoppa approach include peritoneal injuries, injury to the corona mortis or other important vessels (see above), nerve damage, heterotropic ossification, and the formation of incisional hernias [38,39].

For completeness, we would like to mention further approaches as follows: common approaches include the ilioinguinal approach and the pararectus approach for ventral fracture types at the acetabulum, as well as the Kocher–Langenbeck approach for dorsal fracture types. The decision as to which approach to use depends on the classification of the fracture (localization and morphology), the individual patient, and the surgeon’s expertise.

In order to improve the surgical outcome of patients, it can be useful to plan the operation in advance. This can be carried out using three-dimensional planning and CT analysis [40]. Especially for more complicated fractures, this can be a good aid for the operation. Furthermore, navigation technologies are available to improve the outcome, however, provided that an experienced surgeon is involved in the procedure, the accuracy of the screw position with navigation is currently comparable to the results without navigation. Nevertheless, especially for young surgeons, it improves screw positioning and helps overcome the slow learning curve [41]. Further research in the field of navigation and preoperative planning could help improve outcomes and precision.

## 7. Surgical Technique for the Modified Stoppa Approach

An experienced surgeon should perform this approach and teach it to younger surgeons, as serious complications can occur (e.g., bleeding from the corona mortis). Initially, the modified Stoppa approach has a slow learning curve and remains a very technically advanced operation even for experienced surgeons.

**Figure 4:**
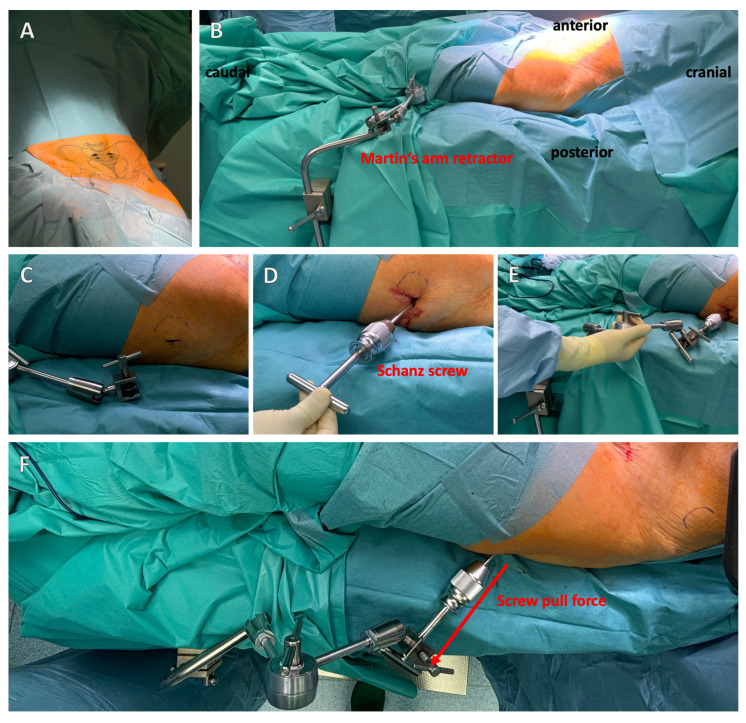
**Positioning of the patient and placement of a Schanz screw with Martin’s arm retractor.** Patients are placed in the supine position (**A**). It is recommended to attach a Schanz screw with permanent tension outside the acetabulum for a better reduction (**B**–**F**). Martin’s arm retractor is suitable for this purpose (**D**–**F**). The red arrow indicates the pull direction (**F**).

For the modified Stoppa approach, the patient is placed in the supine position with the hands extended on either side of the body (Figure 4A). Due to the positioning, the X-ray image converter can be used intraoperatively for the standard anterior–posterior (AP), ala, and obturator images, and, depending on the inclination of the image converter, allows the examination of screws positioned next to the joint [27]. After sterile covering of the patient, a Pfannenstil incision is made, although vertical incisions are also possible. The difference between the incisions is currently the subject of scientific discussion, with a vertical incision being associated with a lower infection rate [42,43].

It is recommended to use femoral protrusion to facilitate anatomical reduction of the acetabulum. This can be achieved using a Schanz screw, which is inserted laterally into the femoral neck and, in the best case, connected to Martin’s arm retractor, allowing the lateral traction to be held permanently (Figure 4B–F).

**Figure 5:**
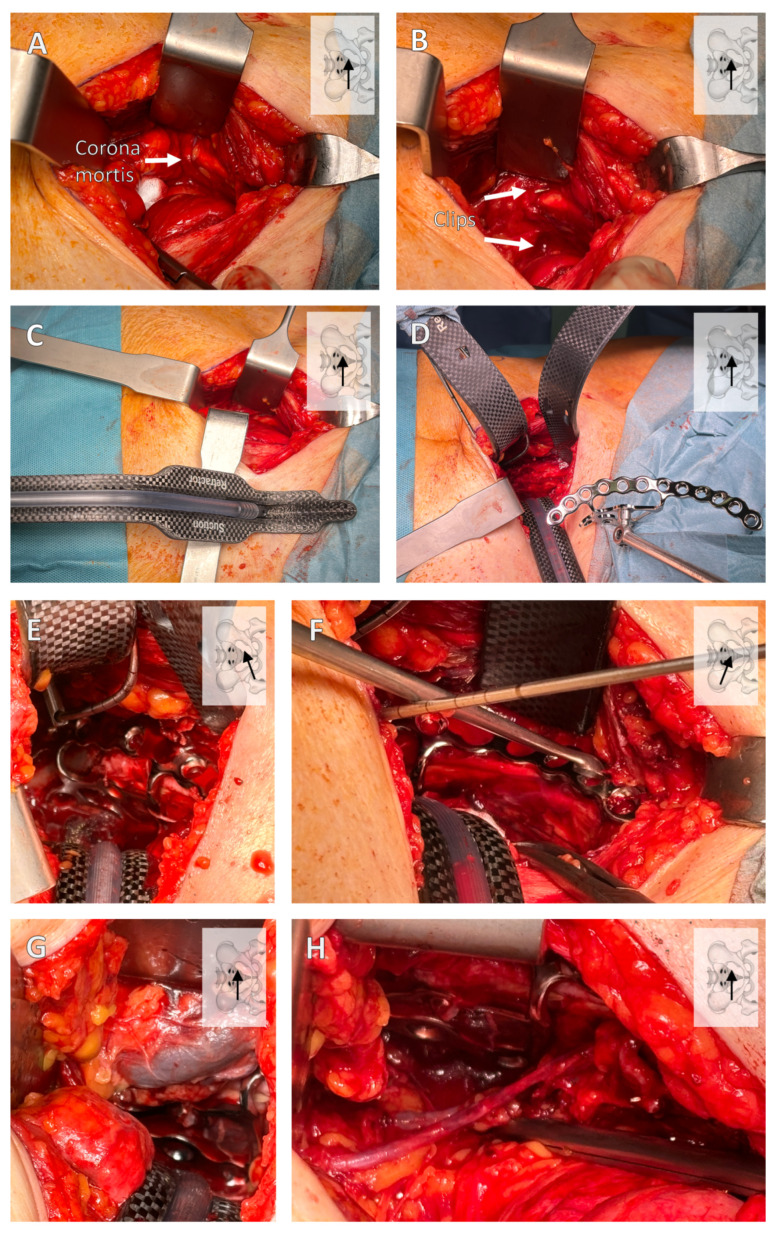
**Important surgical steps of the modified Stoppa approach.** In the retrosymphysial space, a lateral preparation is carried out, where the corona mortis should be visualized (**A**) and, thereafter, clipped (**B**). The carbon retractors are then inserted (**C**,**D**) using a suction retractor (**C**). Placement of the suprapectineal plate with a quadrilateral bearing surface is shown (**D**–**F**). Caution is required when removing the retractors, as important structures such as the external iliac vein or the obturator vessels can be injured (**G**,**H**). The viewing direction is shown at the top left of each image, with the position of the arrow indicating the direction.

After the sharp preparation of the subcutaneous layer, exposure of the fascia, and separation of the fascia in the median line with careful protection of the bladder, the retrosymphysial space is reached. Now, the fascia can be detached from the anterior branch of the pubic bone so that it can be dissected laterally along the branch of the pubic bone. The first important structure is then found here: the corona mortis (Figure 5A). This should now be clipped and cut inwards through the pelvis (Figure 5B). Now, the dissection is continued posteriorly and then laterally along the linea terminalis.

If possible, carbon retractors should be used to visualize the fracture to obtain a better overview of the intraoperative X-ray control of the screws. In addition, the suction retractor has the advantage of aspiration in the depth (Figure 5C).

After visualizing the fracture, the suprapectineal plate can be inserted directly into the parasymphysis (Figure 5D–F). A major advantage of newer plates is addressing the quadrilateral surface with screw options, which can provide a larger surface area and achieve greater stability through the tension of the screws [13,44,45,46].

**Figure 6:**
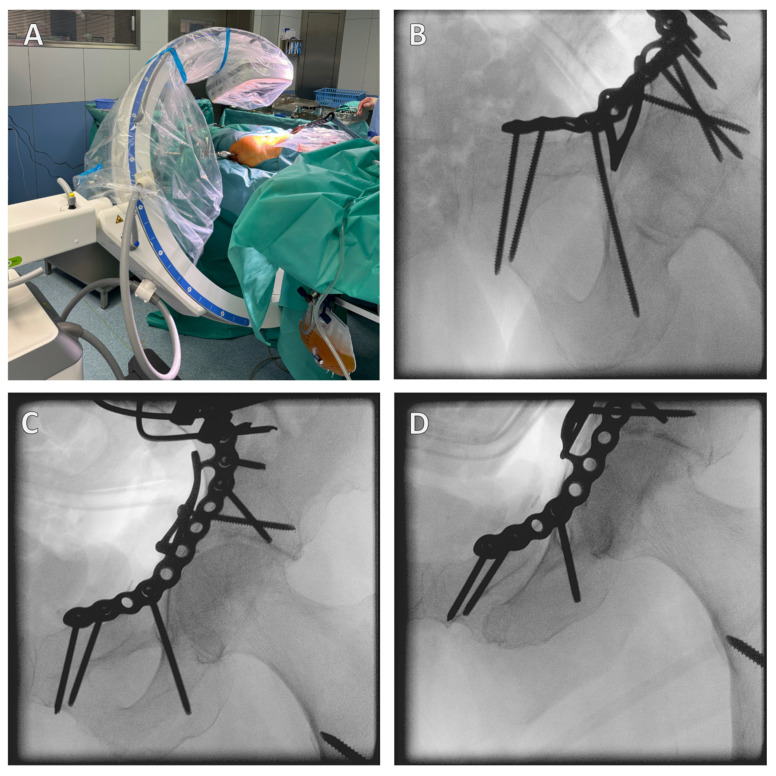
**Intraoperative X-ray control.** It is important to position the patient correctly; a carbon table should be used, and this should be moved out as far as possible so that there is plenty of space to swivel the X-ray C-arm (**A**). The correct screw position is checked in different planes. Here, the infra-acetabular screw is in the correct position, i.e., not through the joint (**B**–**D**).

The correct position of the screws should be checked intraoperatively by X-ray control in the ala/obturator and AP view, which confirms the extra-articular position of the screws (Figure 6). The infra-acetabular screw can be scored in its extra-articular position, confirming the correct position outside the hip joint. Postoperatively, the correct position should be confirmed by CT.

Open reduction and internal fixation (ORIF) of the acetabular fracture is then performed. The correct position of the screws is checked intraoperatively using an X-ray C-arm (Figure 6).

After surgical treatment, a Robinson drain should be placed contralaterally to the drain. An important step after ORIF and drainage is the careful removal of the inserted retractors, as important structures such as the vena iliaca externa can be injured when the retractors are removed (Figure 5G,H).

## 8. Total Hip Arthroplasty for Geriatric Acetabular Fractures

In recent years, total hip arthroplasty (THA) implantation for geriatric acetabular fractures has become of increasing interest to orthopedic and trauma surgeons [47,48]. When special implants are used, the treatment of anterior, posterior, and more complex acetabular fractures shows good results and is currently being discussed as an alternative to ORIF [49].

Currently, there are no precise indications, and scientific consensus needs to be established. However, the first studies conducted have shown a positive outcome and suggest the following indications for THA in acetabular fractures: osteoporotic bone, avascular necrosis of the femoral head, dome impaction, associated injury of the femoral head, and non-rupturable articular communication [48,50].

The advantages of THA are rapid mobilization and significant pain relief, attracting more interest as an alternative to ORIF and non-surgical treatment in geriatric acetabular fractures [47,51]. Complications specific to THA must still be expected: material fatigue, dislocations, periprosthetic femoral fracture, and aseptic loosening. However, it should be mentioned, at this point, that this is currently still the subject of research, and there are no specific recommendations from current professional associations. Further studies should be carried out on this topic.

## 9. Conclusions

Due to the aging demographic and the associated deterioration in bone quality, geriatric acetabular fractures are becoming increasingly common. Currently, geriatric acetabular fractures constitute the majority of acetabular fractures [1,14]. The frequency of acetabular fractures of anterior structures has also increased due to the pathomechanism mentioned above.

Minimally invasive approaches such as the modified Stoppa approach help patients who require surgical treatment achieve good clinical outcomes and rapid mobilization. It is important to ensure that surgeons are well informed about the diagnostics, classification, essential surgical steps, and correct positioning of the X-ray C-arm.

Nevertheless, a high level of surgical expertise is required, and attention must be paid to the following pitfalls: careful preparation of the corona mortis and its clipping, correct positioning of the X-ray imaging system for intraoperative screw correction, and careful removal of the retractors to avoid injuring the vena iliaca externa.

Although THA has shown good results in certain geriatric acetabular fractures, a general recommendation for this treatment cannot be made yet. THA should be further investigated as an alternative surgical option, especially in a direct comparison of the same fracture types with ORIF.

## Figures and Tables

**Table 1:** **AO/OTA classification for acetabular fractures.** The fractures are categorized into three large groups according to articular involvement and column and/or wall fracture: 62A, 62B, and 62C. Subsequently, the subgroups can be categorized according to fracture morphology. ASIS: anterior superior iliac spine.

Type	Type of Fracture Genesis	Subtype
**62A**	Partial articular involvement with isolated fracture of a column and/or wall	62A1.1: Simple fracture
		62A1.2: Multifragmentary fracture
		62A2.1: Through the ischium
		62A2.2: Through the obturator ring
		62A2.3: With associated posterior wall fracture
		62A3.1: With anterior wall fracture
		62A3.2: With high anterior column fracture
		62A3.3: With low anterior column fracture
**62B**	Partial articular involvement with transverse fracture	62B1.1: Infratectal fracture
		62B1.2: Juxtatectal fracture
		62B1.3: Transtectal fracture
		62B2.1: With infratectal transverse component
		62B2.2: With juxtatectal transverse component
		62B2.3: With transtectal transverse component
		62B3.1: Associated with anterior wall
		62B3.2: High anterior column fracture (exits along iliac crest)
		62B3.3: Low anterior column fracture (exits below ASIS)
**62C**	Complete articular involvement with fracture of both columns	62C1: With high anterior column fracture (exits along iliac crest)
		62C2: Low anterior column fracture (exits below ASIS)
		62C3: Involving the sacroiliac (SI) joint

## Data Availability

No patient data were recorded for this review article.
